# Risk of chronic liver disease in post-menopausal women due to body mass index, alcohol and their interaction: a prospective nested cohort study within the United Kingdom Collaborative Trial of Ovarian Cancer Screening (UKCTOCS)

**DOI:** 10.1186/s12889-017-4518-y

**Published:** 2017-06-28

**Authors:** Paul M Trembling, Sophia Apostolidou, Aleksandra Gentry-Maharaj, Julie Parkes, Andy Ryan, Sudeep Tanwar, Matthew Burnell, Ian Jacobs, Usha Menon, William M. Rosenberg

**Affiliations:** 1 Institute for Liver and Digestive Health, Division of Medicine, University College London, Royal Free Hospital, Rowland Hill Street, London, NW3 2PF UK; 2 Gynaecological Cancer Research Centre, University College London Elizabeth Garrett Anderson Institute for Women’s Health, University College London, London, UK; 3 Public Health Sciences and Medical Statistics, Faculty of Medicine, University of Southampton, Southampton, UK; 4 Office of the President and Vice-Chancellor, The University of New South Wales, UNSW Sydney, Sydney, Australia

**Keywords:** Chronic liver disease, Cirrhosis, Alcohol, Body mass index, Obesity

## Abstract

**Background:**

We investigated the risk of chronic liver disease (CLD) due to alcohol consumption and body mass index (BMI) and the effects of their interaction in a prospective cohort study of women recruited to the UKCTOCS trial.

**Methods:**

95,126 post-menopausal women without documented CLD were stratified into 12 groups defined by combinations of BMI (normal, overweight, obese) and alcohol consumption (none, <1–15, 16–20 and ≥21 units/week), and followed for an average of 5.1 years. Hazard ratios (HR) were calculated for incident liver-related events (LRE).

**Results:**

First LREs were reported in 325 (0.34%) participants. Compared to women with normal BMI, HR = 1.44 (95% CI; 1.10–1.87) in the overweight group and HR = 2.25 (95% CI; 1.70–2.97) in the obese group, adjusted for alcohol and potential confounders. Compared to those abstinent from alcohol, HR = 0.70 (95% CI; 0.55–0.88) for <1–15 units/week, 0.93 (95% CI; 0.50–1.73) for 16–20 units/week and 1.82 (95% CI; 0.97–3.39) for ≥21 units/week adjusted for BMI and potential confounders. Compared to women with normal BMI drinking no alcohol, HR for LRE in obese women consuming ≥21 units/week was 2.86 (95% CI; 0.67–12.42), 1.58 (95% CI; 0.96–2.61) for obese women drinking <1–15 units/week and 1.93 (95% CI; 0.66–5.62) in those with normal BMI consuming ≥21 units/week after adjustment for potential confounders. We found no significant interaction between BMI and alcohol.

**Conclusion:**

High BMI and alcohol consumption and abstinence are risk factors for CLD in post-menopausal women. However, BMI and alcohol do not demonstrate significant interaction in this group.

**Trial registration:**

UKCTOCS is registered as an International Standard Randomised Controlled Trial, number ISRCTN22488978. Registered 06/04/2000.

**Electronic supplementary material:**

The online version of this article (doi:10.1186/s12889-017-4518-y) contains supplementary material, which is available to authorized users.

## Background

Chronic liver disease (CLD) is the 5th commonest cause of death in the UK, and the only rising major cause of mortality and morbidity. 60,000 people in England and Wales have cirrhosis [1–3]. Recent data estimates that over 600,000 adults in the USA have CLD, with over half of affected individuals unaware of the diagnosis [4]. Overweight and alcohol consumption are major causes of CLD [5–7]. Non-alcoholic fatty liver disease (NAFLD) can be considered the pathological manifestation in the liver of the metabolic syndrome, of which high BMI is a key feature [8]. NAFLD comprises a spectrum of disease, from steatosis, through inflammation (steatoheaptitis) to fibrosis and cirrhosis. The precise influence of body mass index (BMI) on the risk of liver disease in women, however, is not conclusive and previous studies using smaller subsets of ICD-10 codes to identify liver-related morbidity and mortality may have underestimated the impact of BMI and alcohol [9, 10]. Further, interaction between alcohol and BMI and risk of liver disease is not well understood. Regardless of the etiology of liver disease, the clinicopathological outcome in those who develop CLD is cirrhosis [11] and the there may be common pathways in which alcohol and high BMI damage the liver [12]. A synergistic interaction between steatosis and alcohol consumption in the progression of fibrosis in patients with chronic hepatitis C has been demonstrated in histological studies [13].

Both alcohol consumption and NAFLD are common. Moderate alcohol consumption is associated with decreased mortality, largely due to reduced cardiovascular-related disease, but there are no guidelines related to alcohol use in NAFLD and these factors, in addition to rising levels of liver disease and the high prevalence of excess alcohol consumption, coupled with the worldwide increase in obesity demonstrate the need to further understand the roles of alcohol and BMI and their interaction in CLD.

In a large cohort of women we investigated incidence of CLD and its relationship to alcohol and BMI, and examined the interaction between these two risk factors.

## Methods

### Study population

This prospective cohort study was nested in the United Kingdom Collaborative Trial of Ovarian Cancer Screening (UKCTOCS) [14]. UKCTOCS is a multi-center UK-based randomised controlled trial designed to define the effect of ovarian cancer screening on mortality. Between April 2001 and October 2005, 202,638 post-menopausal women aged 50–74 were recruited in England, Wales and Northern Ireland. Participants were invited at random from 27 local authority registers. Exclusion criteria included bilateral oophorectomy, increased risk of familial ovarian cancer, previous ovarian cancer and active non-ovarian cancer. The trial design and detailed eligibility criteria have been described elsewhere [14–16]. This study is nested within UKCTOCS, comprising of participants in England.

UKCTOCS was approved by the UK North West Multicentre Research Ethics Committee (North West MREC 00/8/34), with site-specific approval from the local regional ethics committees and the Caldicott guardians (data controllers) of the participating primary care trusts. Written informed consent was obtained from all volunteers.

### Exposures

The exposures of interest were BMI and current weekly alcohol consumption. Participants completed a questionnaire at recruitment, which included self-reported height and weight. BMI was calculated (BMI (kg/m^2^) = weight (kg)/(height (m))^2^) and categorised according to the World Health Organisation’s definitions; normal (<25 kg/m^2^), overweight (25- < 30 kg/m^2^) or obese (≥30 kg/m^2^). As there are no existing population estimates for the range of BMI a pragmatic approach was adopted to selecting patients with plausible BMI values. Participants who recorded a height outside the range 140-210 cm, or a weight outside the range 25-200 kg, or where the BMI was outside the range 16–65 kg/m^2^ were excluded.

Via a follow-up questionnaire 3–5 years after randomisation, participants estimated their current alcohol consumption as the number of drinks consumed per week (none, less than 1, 1–3, 4–6, 7–10, 11–15, 16–20 or ≥21 drinks), assuming one drink is a glass of wine, half a pint of beer or cider, or a measure of spirits. Alcohol units were calculated using the convention that one drink is the equivalent of 1 UK unit (10 ml or 8 g of pure alcohol) [17]. Participants were categorised in the following groups; none, <1–15, 16–20 and ≥21 units/week, and those with no alcohol response were excluded.

### Covariates

The follow-up questionnaire asked participants to report known comorbidities including heart disease, hypercholesterolaemia, hypertension and diabetes mellitus, and whether they currently smoked (all categorised as yes/no). Socioeconomic status was estimated using the Index of Multiple Deprivation 2007 (IMD) (continuous variable) [18]. This ascribes a deprivation score to participants based on their postcode, with a higher score indicating higher deprivation.

### Follow up

All participants are followed through a ‘flagging’ study with the NHS Information Centre for Health and Social Care in England and Wales which provided data on cancer registrations and deaths, with diagnosis/cause of death coded according to the International Classification of Diseases, version 10 (ICD-10). 99.98% of UKCTOCS participants were successfully flagged. In addition, hospital inpatient and outpatient episode data for 2001–10 were available through linkage to the Hospital Episodes Statistics (HES) database. Each HES record reports a main diagnosis and up to 19 (inpatient admissions) and 11 (outpatient appointments) further diagnoses and each death record reports the primary death code and additional diagnoses recorded on the death certificate. As HES data were only available for participants in England, only participants in England were included in this study. Women were included in the study from the point of return of questionnaire. Women with known pre-existing liver disease were not included, by excluding those where a code of interest had been registered between recruitment to UKCTOCS and return of questionnaire.

### Outcome

The main outcome measure was first liver-related event (LRE), defined as first presentation of either a hospital admission, outpatient appointment, cancer registration with, or death from, an ICD-10 code of interest. The following codes for liver disease were searched for: K70 (alcoholic liver disease), K73 (chronic hepatitis) and K74 (fibrosis and cirrhosis). These codes are consistent with other UK studies of cirrhosis [1, 9]. We also included K76 (other diseases of liver, including fat) in order to widen the search for liver disease beyond cirrhosis to include fatty liver disease. In addition, codes relating to sequelae of decompensated liver disease were also searched for; I85 (oesophageal varices), Z94.4 (liver transplant) and C22.0 (hepatocellular carcinoma). In addition to ICD-10 codes, death certificates were also searched for any mention of alcoholic liver disease or non-alcoholic fatty liver disease.

### Statistical analysis

Crude incidence rates of first LRE were calculated using person-years of follow-up as the denominators, for each BMI group, each alcohol group and each BMI/alcohol combination. For each participant, person-years of follow-up were accrued from date that the follow-up questionnaire was returned (as this was the date that current alcohol use was ascertained), to the censorship date (February 1, 2013), date of first presentation with LRE, or death from any other cause. Participants who experienced a LRE at any time from randomisation to return of questionnaire were excluded.

#### Separate influences of BMI and of alcohol on incident liver disease

Cox proportional hazards models were used to calculate hazard ratios (HRs) of first LRE in three categories of BMI using normal BMI as the reference. Similar analysis was performed for alcohol with no alcohol consumption as the reference. The proportional hazards models were adjusted for BMI, or alcohol respectively.

All potential confounding risk factors (smoking, IMD, hypertension, heart disease, hypercholesterolaemia, diabetes) were included individually in a Cox model to calculate univariate HRs for LREs, to guide their utility in the models evaluating risk due to BMI and alcohol.

#### Influences of combinations of BMI and alcohol

HRs were calculated for twelve BMI and alcohol combinations using the normal BMI/no alcohol consumption category as the reference, adjusted for potential confounders with significant HRs for LRE, and then adjusted only for factors associated with the metabolic syndrome (hypertension, hypercholesterolemia, heart disease and diabetes). The proportional hazards assumption was checked by examining the log minus log plot.

#### Interaction between alcohol and BMI

Interaction between alcohol using several thresholds and BMI (as a continuous variable) was analysed by calculating the interaction term from the Cox regression models.

All analyses were performed using SPSS (version 19, SPSS Inc., Chicago, IL, USA) and STATA statistical software (StataCorp 2007. Release 10. College Station, TX, USA: StataCorp LP).

## Results

**Fig. 1:**
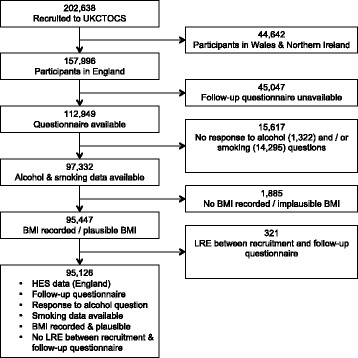
The composition of the final study cohort and its derivation from the UKCTOCS cohort

Of the 157,996 UKCTOCS participants resident in England, 62,870 were excluded including 321 women who experienced an LRE or died between recruitment and return of questionnaire and 14,295 (9%) with no data on smoking. The final cohort comprised 95,126 participants (Fig. 1).

Baseline characteristics are shown in Table 1. 97.1% of the participants were white. 36% were smokers. 55% were either overweight (37%) or obese (19%). 23.4% reported drinking no alcohol and 1.5% reported drinking more than 21 units/week. Increasing BMI correlated with increased reporting of hypertension, heart disease, hypercholesterolaemia and diabetes.

**Table 1:** Baseline characteristics, number of first events according to BMI category and in all participants, and hazard ratios for LRE for potential confounders (continuous^a^ and categorical^b^ variables)

Characteristic	BMI category (kg/m^2^)			All participants	Hazard ratio (95% confidence intervals)
	<25	25 - < 30	≥30		
Total, *n* (%)	42,452 (44.6)	35,073 (36.9)	17,601 (18.5)	95,126	
Recruitment age, median (years)	60.0 (50–74)	61.0 (50–74)	60.0 (50–74)	60.0 (50–74)	1.01^a^ (0.99–1.02)
Smoker, *n* (%)	14,740 (34.7)	12,616 (36.0)	6621 (37.6)	33,977 (35.7)	1.89^b^ (1.52–2.35)
Hypertension, *n* (%)	9477 (22.3)	12,116 (34.5)	8440 (48.0)	30,033 (31.6)	1.38^b^ (1.11–1.73)
Heart disease, *n* (%)	1721 (4.1)	2086 (5.9)	1416 (8.0)	5223 (5.5)	2.17^b^ (1.53–3.06)
Hypercholesterolemia, *n* (%)	8001 (18.8)	9148 (26.1)	5440 (30.9)	22,589 (23.7)	1.68^b^ (1.33–2.11)
Diabetes, *n* (%)	836 (2.0)	1689 (2.6)	2263 (12.9)	4788 (5.0)	2.76^b^ (1.99–3.83)
IMD, mean	17.0	18.7	21.3	18.5	1.09^a^ (1.01–1.03)
Alcohol consumption (units/week)					
None	8479 (20.0)	8189 (23.3)	5547 (31.5)	22,215 (23.4)	1^b^ (reference)
< 1–15	31,811 (74.9)	25,324 (72.2)	11,473 (65.2)	68,608 (72.1)	0.64^b^ (0.51–0.82)
16–20	1448 (3.4)	1067 (3.0)	366 (2.1)	2881 (3.0)	0.82^b^ (0.44–1.53)
≥ 21	714 (1.7)	493 (1.4)	215 (1.2)	1422 (1.5)	1.66^b^ (0.89–3.09)
Alcohol consumption (units/week)	Number of first LREs				
None	23	36	42	101	
< 1–15	71	77	55	202	
16–20	17	10	3	11	
≥ 21	4	5	2	11	
Total	102	123	100	325	

*BMI* body mass index, *IMD* Index of Multiple Deprivation, *LRE* liver-related event

Three hundred twenty five (0.34%) women experienced a first LRE over a total of 509,561 person-years of follow-up (mean 5.1 years), equivalent to 0.64 first events per 1000 person-years (3.3 per 1000 women over 5 years). The most common ICD-10 code signaling a first presentation of LRE was K76 (Additional file 1: Table S1). Only 763 (0.8%) of participants were underweight (BMI <18·5 kg/m^2^) and in this group there were only 4 LREs, therefore this group was combined with the normal BMI group. 1237 (7% of the obese group) women could be classified as morbidly obese (BMI ≥ 40 kgm^−2^) and in this group, the event rate was highest (1.98 events per 1000 person years (95% CI; 1.05–3.38)). There were 2713 (2.9%) deaths from any cause.

### Risk of liver-related events due to potential confounders

Other covariates also demonstrated independent association with liver-related events (Table 1). Significant HRs were seen with smoking, hypertension, heart disease, hypercholesterolaemia, diabetes and IMD.

### BMI and risk of liver-related events

Crude rates of LREs increased with rising BMI. HRs for LRE were significantly higher in both overweight (1.44, 95% CI; 1.10–1.87) and obese categories (2.25, 95% CI; 1.70–2.97) compared to the normal BMI group. A fully adjusted model is presented incorporating adjustment for confounders with significant HRs (Table 2).

**Table 2:** Event rates and adjusted hazard ratios of first liver-related events, according to BMI category and according to alcohol category

BMI and alcohol categories	First event rate per 1000 person years (95% confidence intervals)	Hazard ratio (95% confidence intervals)^a^	Hazard ratio (95% confidence intervals)^b^
BMI category (kg/m^2^)			
< 25	0.45 (0.4–0.5)	1 (reference)	1 (reference)
25 - <30	0.65 (0.5–0.8)	1.44 (1.10–1.87)	1.31 (1.01–1.72)
≥ 30	1.06 (0.9–1.3)	2.25 (1.70–2.97)	1.85 (1.38–2.48)
Alcohol category (units/week)			
None	0.86 (0.7–1.0)	1 (reference)	1 (reference)
< 1–15	0.55 (0.5–0.6)	0.70 (0.55–0.88)	0.78 (0.61–1.00)
16–20	0.68 (0.3–1.2)	0.93 (0.50–1.73)	0.97 (0.52–1.82)
≥ 21	1.37 (0.7–2.5)	1.82 (0.97–3.39)	1.83 (0.97–3.44)

^a^Adjusted for BMI (continuous variable) or alcohol category as appropriate ^b^Adjusted for BMI (continuous variable) or alcohol category as appropriate and smoking, hypertension, heart disease, hypercholesterolaemia, diabetes and IMD

### Alcohol consumption and risk of liver-related events

The rate of LRE was lowest in the group drinking <1–15 units weekly and increased with abstinence and increasing alcohol use. This tendency towards a “J-shaped” relationship between LRE and alcohol consumption was preserved after adjustment for BMI, with lowest HRs in the <1–15 units/week group, although the there was no statistically significant difference between the HRs for this group and the reference group. A fully adjusted model is shown, adjusted for variables with significant HRs for LRE (Table 2).

In the group reporting no alcohol consumption the proportion of LREs that were alcohol-related was 3.96% compared to 11.16% in those drinking any alcohol.

### Risk of liver-related events in participants grouped in to combinations of BMI and alcohol use

Participants were grouped according to combinations of BMI and alcohol consumption. Table 3 shows the rates of LRE in each group. The fully adjusted Cox model shows that the lowest risk is in those with normal BMI consuming <1–15 units/week. Within the normal BMI group, abstinence or drinking >16 units/week increases the risk of LRE, although there are wide confidence intervals.

**Table 3:** Event rates and hazard ratios of first liver-related event according to various BMI and alcohol combinations

BMI category (kg/m^2^)	Alcohol category (units/week)			
	None	<1–15	16–20	≥21
	First event rate per 1000 person years (95% confidence intervals)			
<25	0.52 (0.3–0.8)	0.42 (0.3–0.5)	0.50 (0.1–1.3)	1.00 (0.3–2.6)
25 - <30	0.83 (0.6–1.2)	0.57 (0.4–0.7)	0.84 (0.3–2.0)	1.81 (0.6–4.2)
≥30	1.43 (1.0–1.9)	0.88 (0.7–1.1)	0.98 (0.1–3.5)	1.68 (0.2–6.1)
	Hazard ratio (95% confidence intervals) adjusted for smoking, hypertension, heart disease, hypercholesterolaemia, diabetes and IMD			
<25	1 (reference)	0.91 (0.56–1.47)	1.03 (0.35–2.99)	1.93 (0.66–5.62)
25 - <30	1.46 (0.85–2.50)	1.34 (0.71–1.83)	1.61 (0.61–4.26)	3.32 (1.25–8.81)
≥30	2.28 (1.35–3.86)	1.58 (0.96–2.61)	1.67 (0.39–7.15)	2.86 (0.67–12.21)
	Hazard ratio (95% confidence intervals) adjusted for hypertension, heart disease, hypercholesterolemia and diabetes			
<25	1 (reference)	0.85 (0.53–1.37)	1.07 (0.37–3.09)	2.13 (0.74–6.17)
25 - <30	1.51 (0.89–2.55)	1.11 (0.69–1.76)	1.74 (0.66–4.57)	3.69 (1.40–9.72)
≥30	2.35 (1.40–3.95)	1.59 (0.97–2.60)	1.89 (0.44–8.01)	3.16 (0.74–13.41)

Among overweight and obese women, the nadirs of risk were in the <1–15 units/week groups, and as in the normal BMI group, the risk was highest in the highest alcohol group (HR 3.32, 95% CI; 1.25–8.81; and HR 2.86, 95% CI; 0.67–12.21 respectively).

To estimate the effect of cardiovascular disease and diabetes on the morbidity associated with fatty liver disease, HRs were adjusted for confounding factors associated with the metabolic syndrome. When elements of the metabolic syndrome were controlled for, risk of LRE attributable to heavier drinking increased. This suggests that the risk of liver disease attributable to BMI in patients with, or at risk of, metabolic syndrome is not entirely accounted for by hypertension, heart disease, hypercholesterolemia or diabetes, but may be partly attributable to steatosis itself.

When separated by BMI group, the trend to a “J-shaped” relationship of risk of LRE remains in all BMI groups, with risk highest in the abstainers and heavier drinkers, compared to those in the <1–15 units/week alcohol groups.

### Interaction between alcohol and BMI

Interaction terms were calculated for BMI (continuous) and alcohol, using thresholds for high alcohol of ≥16 units/week and ≥21 units/week. There was no significant interaction between BMI and high alcohol use. Similarly, no interaction was seen with BMI and any alcohol use.

## Discussion

### Main findings

The most striking finding of this study is the risk of liver disease associated with overweight/obesity in post-menopausal women. While the association between alcohol consumption and CLD is well established, there is still much to characterise in the natural history of non-alcoholic fatty liver disease (NAFLD) [3]. Furthermore the study supports the adverse impact of heavy drinking compounding the effects of overweight and obesity. Strategies for preventing and detecting liver disease should be developed accommodating these findings.

This study suggests that in women aged 50–74, those consuming <1–15 units/week are at lowest risk of liver disease. Those drinking 16–20 units/week are only marginally more at risk. The UK Institute of Alcohol Studies defines hazardous drinking as more than 14 units/week and harmful drinking as >35 units/week which would be consistent with the observations in our study population.

Those that are overweight or obese have an increased risk of liver disease. Women of normal BMI who drink <1–15 units/week are at lowest risk, compared to those who drink more or who abstain. It is possible, however, that some abstainers had previously been heavy drinkers. This is supported by our data showing that 4% of LREs in the abstainers were alcohol related.

When combinations of risk are considered, compared to a baseline of normal BMI and abstinence, higher BMI (≥30 kg/m^2^) confers a greater risk than higher alcohol consumption (≥21 units/week). The highest risk is in those who are overweight or obese and drink the most alcohol.

After adjustment for confounding due to metabolic risk factors, HRs in the two highest alcohol categories increased in all BMI groups, suggesting that these factors may contribute to the risk of CLD. It is biologically plausible that diabetes, hypercholesterolaemia and hypertension may contribute to liver disease over and above that caused by fatty liver disease and alcoholic liver toxicity. The corollary is that obesity can cause liver morbidity and mortality in the absence of the metabolic syndrome, providing evidence that case ascertainment cannot be restricted to overweight or obese patients with features of the metabolic syndrome and challenging the “two hit” and “three hit” hypotheses [19].

### Strengths and limitations

Strengths of this study include the size and duration of follow-up, the prospective design and the independence of the data capture for outcomes. This study was also able to adjust for confounding factors, which has not been possible in other cross-sectional studies. In an effort to capture all morbidity and mortality attributable to liver disease, rather than just cirrhosis, we selected ICD-10 codes that encompass a clinically relevant group of diseases including codes for CLD and those relating to the consequences of decompensated liver disease. This was designed to maximise the ability to detect liver disease.

Limitations include reliance on self-reporting of alcohol consumption, co-morbidities, height and weight, which may be a factor in the wide confidence intervals seen for all HR estimates. However, good reliability of self-reporting height and weight [20–24], and alcohol [25–27], has been demonstrated in other studies.

Height and weight were reported at recruitment, and alcohol consumption reported later, on the follow-up questionnaire. Participants were asked to report current alcohol use, rather than lifetime patterns. Changes in drinking patterns would not have been identified, and this method of data collection may fail to identify episodic (“binge”) drinkers. We used the convention that one drink is equivalent to 1 unit of alcohol. However assumptions about alcohol content are difficult to make as measures of volume are likely to vary depending on where the alcohol is consumed, and the alcohol content of drinks continues to change. There is evidence that the number of units in alcoholic drinks in the UK have been undercounted [28], however we have used the standard 1 drink = 1 unit as this remains a widely used convention, particularly in public health promotion.

Reliance on ICD-10 to define events may result in errors due to mis-coding. We used additional codes to those used to define cirrhosis in order to maximize the capture of cases, but these may also be subject to mis-coding. We attempted to reduce the risk of non-coding of events by using 3 independent sources, and in the case of death certification also used hand searching of key words in the text of death certificates. Also, the HES database may not capture some areas of healthcare, for example the private sector. The number of LREs that included ICD-10 Z94.4 is surprising (Additional file 1: Table S1). This may be because participants with liver transplants are engaged in hospital care and are easily identified and coded.

Only post-menopausal women aged 50–74 were included with 97% being white. The loss to follow up rate in UKCTOCS was very small (0.02%). The acceptance rate was 23%. However, despite attempts to ensure that UKCTOCS was representative of the general population [15] there was a ‘healthy volunteer effect’ [29] on both overall and cause-specific mortality, which may have an effect on the generalisability of findings [18]. Although the health section of the follow-up questionnaire did not specifically ask about liver disease, we excluded those who had a code of interest recorded between recruitment to UKCTOCS and the start of this study. However, exclusion of all participants with known CLD could not be guaranteed.

It is unlikely that viral hepatitis made a significant contribution to LRE based on low prevalence in the demographic of women in this study [30]. During the follow-up period in our study, only 21 (0.02%) of participants had a code for viral hepatitis recorded.

### Other studies

A number of studies have demonstrated a reduced risk of liver disease in patients with NAFLD who consume low or moderate amounts of alcohol [31–33], and it has been suggested that these levels of alcohol use may be associated with beneficial effects of insulin sensitivity in post-menopausal women [34]. However, at higher extremes of BMI and alcohol use, data is not conclusive. Previous studies have attributed a lower incidence of CLD to BMI and alcohol, and as expected a lower incidence of CLD when only alcoholic cirrhosis is examined [35]. However these have relied on cirrhosis codes alone, ignoring complications characterising decompensated cirrhosis that are indicative of CLD and clearly associated with BMI and alcohol included in the present study. This study is in broad agreement with some other studies including the National Health and Nutrition Examination Survey (NHANES) [6] which found increasing risk with both increasing BMI and alcohol, but no excess risk in overweight or obese drinkers or in abstainers. A Scottish prospective study reported increasing risk with increasing BMI in men, but not in women [10]. A sub-analysis of men found the lowest risk of CLD in abstainers with normal BMI with a supra-additive interaction between BMI and alcohol [36]. The UK-based Million Women Study [9] used a limited range of ICD-10 codes to identify cirrhosis and reported a rate of hospital admission or death from liver disease less than half that found in our study. However, as in our study, highest risk was in overweight or obese women consuming the most alcohol. In a study of patients with a history of alcohol excess who were admitted to hospital with an alcohol-related problem, risk of cirrhosis was twice as high among the overweight group as those with normal BMI [37]. A recent prospective study of 107,735 middle-aged males used self-reported BMI and alcohol use to assess liver-related mortality ascertained form record linkage, using ICD-10 codes K70-K76, demonstrating a U-shaped relationship between alcohol and mortality and BMI and mortality. Although there was evidence of synergy between low BMI and high alcohol, as in our study there was no evidence of interaction between high BMI and high alcohol use [38].

Our finding of increased risk in abstainers has precedent but is controversial. Previous studies have demonstrated the “J-shaped” relationship between alcohol and risk of mortality [39–42] or CLD [43, 44]. Some prospective studies have found that men but not women abstainers were at increased risk [9, 44], in contrast to the present study that provides a more comprehensive insight into the effects of weight and alcohol. Using raised aminotransferase levels to diagnose suspected NAFLD in men and women in NHANES the highest risk was seen in non-drinkers compared to modest drinkers [45], and in biopsy-proven NAFLD, moderate drinkers had lower risk of steatohepatitis compared to non-drinkers [46]. A prospective Danish study investigating risk of alcohol-related cirrhosis in over 30,000 participants found a dose-dependent increase in risk of cirrhosis with increasing alcohol in women, rather than a “J-shaped” relation which they observed in males [43].

We have confirmed this relationship with risk of CLD in our cohort, and also have demonstrated that the trend towards a “J-shape” relationship remains, irrespective of BMI group.

The increased risk of alcoholic cirrhosis in abstainers compared to light drinkers may be due, in part, to this group containing previous drinkers who raise the overall risk in the abstainer group, rather than due to a true protective effect of alcohol in the light drinkers. One prospective study [35] demonstrated the loss of the “J-shaped” curve when lifetime abstainers were separated from current abstainers. In a small study of patients with biopsy-proven NAFLD, a comprehensive alcohol history was obtained and found to be higher than the original estimate at diagnosis in some patients, suggesting that some of these patients may have had alcohol-related liver disease rather than NAFLD [47].

We found alcohol-related LREs in abstainers (although at less than half the rate seen in drinkers) which, although may partly be a function of miscoding, provides further evidence that this group comprises some ex-drinkers.

Interaction between higher levels of alcohol consumption and NAFLD may result in greater risk of liver disease. A study measuring aminotransferase activity found that increased BMI potentiates the harmful effect of alcohol on the liver [48]. Increased aminotransferase levels were associated with higher alcohol consumption and BMI. In those with normal BMI there was no association between alcohol and raised aminotransferase levels, but in the overweight and obese groups, alcohol increased risk of elevated aminotransferases. A study of an older population also found risk of elevated aminotransferases with increased BMI and increased alcohol consumption (with lowest risk in abstainers), and a large synergistic effect in the obese group consuming more than three drinks/day [49]. This group also examined the risk of hepatocellular carcinoma in people with chronic hepatitis B, finding synergism between obesity and alcohol [50, 51].

### Implications

Our results suggest a substantial influence of both elevated BMI and alcohol on risk of CLD. Although no significant interaction between BMI and alcohol was seen and this lack of synergy is reassuring, the compelling risk in the overweight and obese groups adds to the evidence that rising BMI and increasing alcohol use are risk factors for liver disease among women.

By considering the clinical consequences of liver disease beyond the diagnosis of cirrhosis we revealed a greater burden of disease than previously recognised. Currently much CLD goes undiagnosed until complications of cirrhosis result in serious morbidity and mortality. Earlier identification of those at risk could avert illness and reduce costs by targeted interventions. While the risks associated with heavy alcohol consumption are frequently publicised these data emphasise the importance of disseminating awareness of the risks of liver disease associated with BMI, particularly in light of the growing prevalence of overweight and obesity throughout the world [52]. Public health policy and health education and awareness campaigns should take these facts into account.

## Conclusion

This study of post-menopausal women suggests that elevated BMI and high alcohol intake are independent risk factors for liver disease. Strategies for detecting liver disease and public health strategy should recognise the importance of BMI as well as alcohol when confronting the growing burden of liver disease.

## Additional file

Additional file 1: Figure S1. Crude rates of first liver-related events (per 1000 person years) according to a) BMI category and b) alcohol consumption category over mean follow-up of 5.1 years. **Table S1.** ICD-10 codes and death certificate text of first LREs. (DOCX 120 kb)

## Abbreviations

BMI: Body mass index
CLD: Chronic liver disease
HES: Hospital episode statistics
HR: Hazard ratio
ICD-10: International classification of disease 10th revision
IMD: Index of multiple deprivation
LRE: Liver-related event
NAFLD: Non-alcoholic fatty liver disease
NHANES: National health and nutrition examination survey
UKCTOCS: United Kingdom Collaborative Trial of Ovarian Cancer Screening
