# Supplementary material for: Risk of chronic liver disease in post-menopausal women due to body mass index, alcohol and their interaction: a prospective nested cohort study within the United Kingdom Collaborative Trial of Ovarian Cancer Screening (UKCTOCS)
Source: BMC Public Health. 2017 Jun 28;17:603. doi: 10.1186/s12889-017-4518-y (PMC5490218; doi:10.1186/s12889-017-4518-y)
Supplement: Additional file 1: Figure S1. — Crude rates of first liver-related events (per 1000 person years) according to a) BMI category and b) alcohol consumption category over mean follow-up of 5.1 years. Table S1. ICD-10 codes and death certificate text of first LREs. (DOCX 120 kb) [file 12889_2017_4518_MOESM1_ESM.docx]

**RISK OF CHRONIC LIVER DISEASE IN POST-MENOPAUSAL WOMEN DUE TO BODY MASS INDEX, ALCOHOL AND THEIR INTERACTION: A PROSPECTIVE NESTED COHORT STUDY WITHIN IN THE UNITED KINGDOM COLLABORATIVE TRIAL OF OVARIAN CANCER SCREENING (UKCTOCS)**

**ADDITIONAL FILE**

Contents

**Figure S1** Crude rates of first liver-related events (per 1000 person years) according to a) BMI category and b) alcohol consumption category over mean follow-up of 5.1 years

**Table S1 CD-10 codes and death certificate text of first LREs**

**Figure S1**

**Crude rates of first liver-related events (per 1000 person years) according to a) BMI category and b) alcohol consumption category over mean follow-up of 5.1 years**

The change in crude rates for first liver related events associated with increasing BMI and alcohol consumption are depicted. The rate of LRE rises with increasing BMI but follows a “J” or “U” shaped relationship with alcohol consumption in which abstinence is associated with a greater risk than moderate consumption (<1-15 units/week).

a)


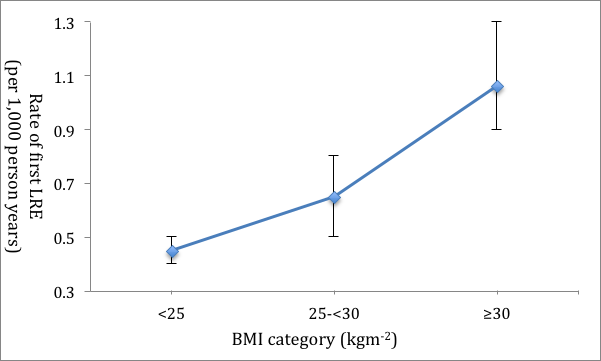


b)


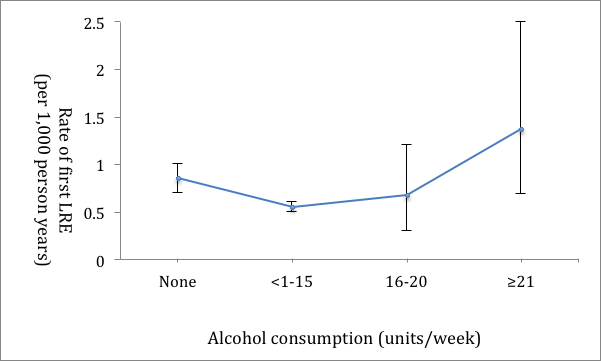


**Table S1**

**ICD-10 codes and death certificate text of first LREs**

The number of codes / death certificate text results is higher than the number of LREs (325) as some participants had more than one code when presenting with first LRE

| **Source** | **Code or text** | **Number of participants (% of those with LRE)** |
| --- | --- | --- |
| Hospital admission | K70 | 15 (4.6) |
|  | K73 | 9 (2.8) |
|  | K74 | 45 (13.8) |
|  | K76 | 183 (56.3) |
|  | C22.0 | 7 (2.2) |
|  | I85 | 12 (3.7) |
|  | Z94.4 | 33 (10.2) |
| Outpatient appointment | K74 | 1 (0.3) |
|  | Z94.4 | 11 (3.4) |
| Cancer registration | C22.0 | 12 (3.7) |
| Death certificate | K70 | 6 (1.8) |
|  | K74 | 7 (2.2) |
|  | K76 | 10 (3.1) |
|  | C22.0 | 2 (0.6) |
|  | Mention of alcoholic liver disease | 8 (2.5) |
|  | Mention of non-alcoholic fatty liver disease | 8 (2.5) |
